# A comparison of strategies for generating artificial replicates in RNA-seq experiments

**DOI:** 10.1038/s41598-022-11302-9

**Published:** 2022-05-03

**Authors:** Babak Saremi, Frederic Gusmag, Ottmar Distl, Frank Schaarschmidt, Julia Metzger, Stefanie Becker, Klaus Jung

**Affiliations:** 1 Institute for Animal Breeding and Genetics, University of Veterinary Medicine Hannover, Foundation, Hannover, Germany; 2 Institute for Parasitology, University of Veterinary Medicine Hannover, Foundation, Hannover, Germany; 3 Biostatistics Department, Institute for Cell Biology, Leibniz University Hannover, Hannover, Germany; 4 RG Development and Disease, Veterinary Functional Genomics, Max-Planck-Institute for Molecular Genetics, Berlin, Germany

**Keywords:** Transcriptomics, Bioinformatics, High-throughput screening

## Abstract

Due to the overall high costs, technical replicates are usually omitted in RNA-seq experiments, but several methods exist to generate them artificially. Bootstrapping reads from FASTQ-files has recently been used in the context of other NGS analyses and can be used to generate artificial technical replicates. Bootstrapping samples from the columns of the expression matrix has already been used for DNA microarray data and generates a new artificial replicate of the whole experiment. Mixing data of individual samples has been used for data augmentation in machine learning. The aim of this comparison is to evaluate which of these strategies are best suited to study the reproducibility of differential expression and gene-set enrichment analysis in an RNA-seq experiment. To study the approaches under controlled conditions, we performed a new RNA-seq experiment on gene expression changes upon virus infection compared to untreated control samples. In order to compare the approaches for artificial replicates, each of the samples was sequenced twice, i.e. as true technical replicates, and differential expression analysis and GO term enrichment analysis was conducted separately for the two resulting data sets. Although we observed a high correlation between the results from the two replicates, there are still many genes and GO terms that would be selected from one replicate but not from the other. Cluster analyses showed that artificial replicates generated by bootstrapping reads produce it p values and fold changes that are close to those obtained from the true data sets. Results generated from artificial replicates with the approaches of column bootstrap or mixing observations were less similar to the results from the true replicates. Furthermore, the overlap of results among replicates generated by column bootstrap or mixing observations was much stronger than among the true replicates. Artificial technical replicates generated by bootstrapping sequencing reads from FASTQ-files are better suited to study the reproducibility of results from differential expression and GO term enrichment analysis in RNA-seq experiments than column bootstrap or mixing observations. However, FASTQ-bootstrapping is computationally more expensive than the other two approaches. The FASTQ-bootstrapping may be applicable to other applications of high-throughput sequencing.

## Introduction

High-throughput sequencing of RNA samples (RNA-seq) has become the standard for generating gene expression profiles of biological samples with a wide range of examples in biomedical research^1–3^. Due to the overal costs of an RNA-seq experiment, technical replicates are usually omitted, especially in observational studies which already include a large number of biological replicates. The general need for technical replicates has been discussed controversely in the last years. While, for example, a high reproducibility of RNA-seq analyses has been reported, the same study points at cases were technical replicates could improve the statistical power of an experiment^4^. McIntyre et al.^5^ reported that reproducibility also depends on the size of coverage of sequencing reads across the reference genome. It has also been found that technical replicates can be useful to detect potential lane effects of the flow cell^6^, the solid support of the RNA material within the sequencing machine. Moreover, Li et al.^7^ argue for the general need of measuring the reproducibility of high-throughput experiments, since many studies have shown changes in the ranking of selected features (i.e., genes). Such changes can finally lead to different biological interpretation, or to different outcomes of subsequent analyses, e.g. gene-set enrichment analysis^8,9^.

To circumvent to obstacle of additional costs for technical replicates, we study and compare three different approaches to generate replicates of RNA-seq experiments computationally. The first approach has been presented in the context of DNA microarray experiments and takes bootstrap samples from the columns of the gene expression matrix^10^. This technique does not generate new expression values but a new permutation of existing data. Thus, a replicate of the whole experiment and not for individual samples is generated. The second approach uses bootstrap samples from the sequencing reads stored in the individual FASTQ-files. Bootstrapping from a set of sequencing reads has also already been proposed in the context of transcriptomics^11^ and also metagenomics^12^. This approach generates new expression levels for each biological sample that can be regarded as an artificial technical replicate. Finally, we include the approach of mixing observations by using a weighted mean over the columns of the expression matrix, a typical technique for data augmentation in machine learning^13^. Usually, one expects in an experiment that the correlation of data from the same biological sample (e.g. in the form of a technical replicate) is larger than the correlation between data from biologically independent samples. Therefore, by mixing the data of biologically independent samples, this approach appears more appropriate to generate artificially biological replicates. In the following, we denote the three approaches by FB (approach 1, FASTQ-boostrapping), CB (approach 2, column bootstrapping), and MO (approach 3, mixing observations). By their nature, the CB and MO approach do not produce technical replicates for individual samples, but they still may be suited to study the reproducibility of an RNA-seq experiment.

Here, we compare the three approaches of artificially generating replicates of RNA-seq experiments on a data example as a case study produced by an experiment in our own labs. The experiment consists of infected and non-infected samples each subjected twice to RNA-seq, i.e. with two real technical replicates. The two resulting data sets are denoted by R1 and R2 in the following. We observe how well analytical results obtained from artificial replicates generated from data of R1 fit to the results obtained from data of the true technical replicate R2, and vice versa. Comparisons are done on the level of it p values and log fold changes from differential expression analysis and subsequent GO term enrichment analysis. The comparison is mainly done explorative looking at similarities between results from true and artificially generated data. We also compare the gene-wise dispersion estimates for biological and technical variance in the real and artificial data.

## Methods

**Figure 1:**
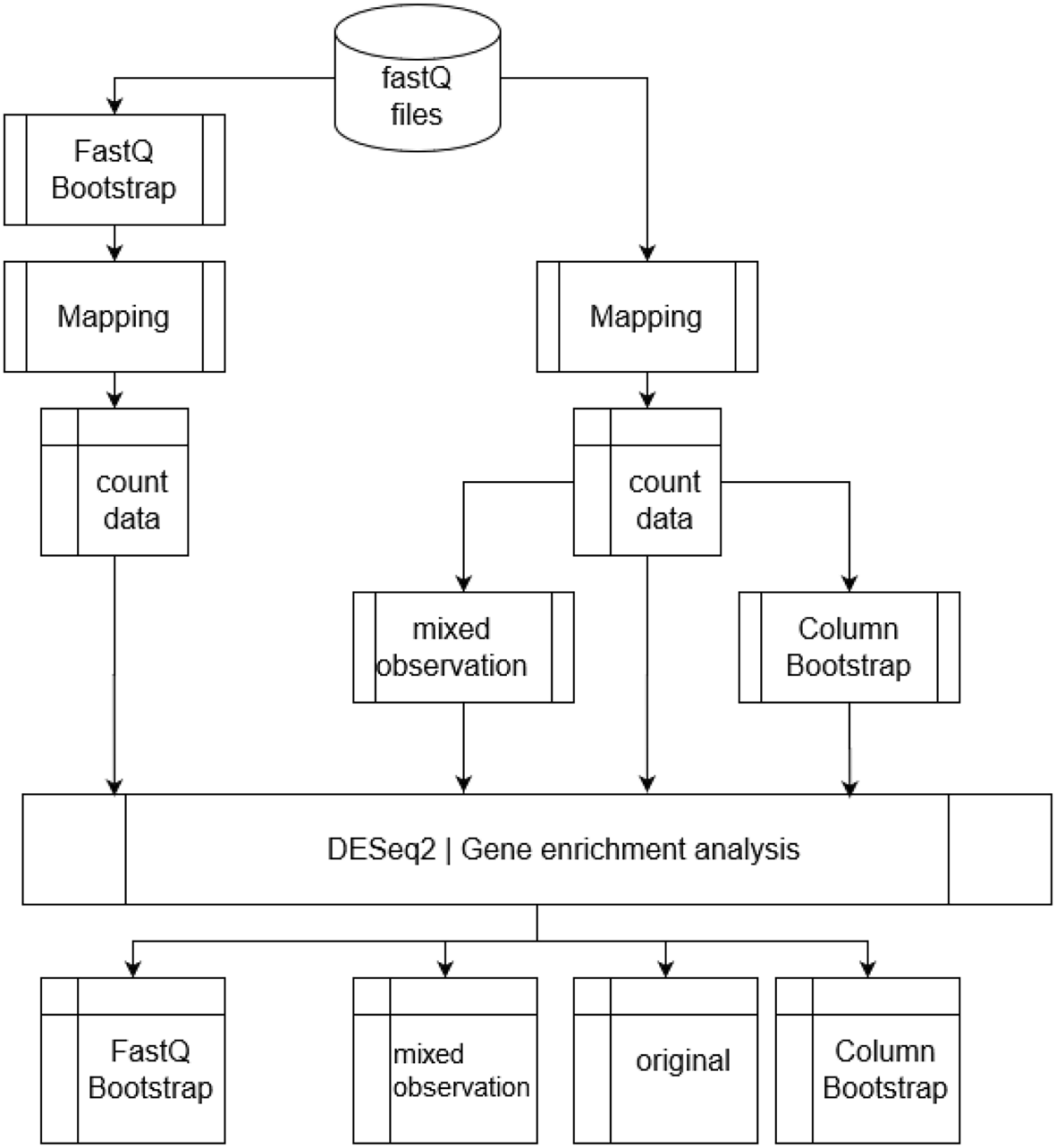
Workflow of generating replicates by the different strategies. While the MO and CB approach are based on the read count data obtained after mapping, the FB approach starts directly with the reads from the FASTQ-files. Finally, the original results of differentially expression analysis and gene-set analysis are compared with the results obtained with the artificial replicates. The diagram was drawn using the software ‘diagrams’ (version 16.2.7, www.diagrams.net).

In this section, we detail first the three different approaches for generating artificial replicates of individual samples or replicates of the whole experiment (Fig. 1). In addition to the computational methods, we present the infection data set, that we use as a case study to demonstrate and compare the computational methods.

### Data processing, mapping, differential expression and enrichment analysis

Raw FASTQ-files were processed by quality-based trimming using Trimmomatic^14^ and mapped to the mouse reference genome (Version GRCm38 from https://www.ncbi.nlm.nih.gov/) using STAR^15^. Each differential expression analysis was performed by the R-package DESeq2^3^ to generate lists of it p values and log fold changes. Raw it p values were adjusted to control a false discovery rate of $5\%$ by the method of Benjamini and Hochberg^16^. GO term enrichment analysis was performed based on the it p value lists from differential expression analysis using Enrichr^17^. Differential expression and enrichment analyses were performed separately for data sets R1 and R2.

### Bootstrapping columns of the expression matrix (CB approach)

After mapping the reads of each original FASTQ-file to the reference genome, a matrix of read counts is generated which contains *d* genes in its rows and *n* samples in its columns. This is similar to expression matrices obtained in DNA microarray experiments, except for the difference that microarray experiments generate fluorescence values as measure of gene expression. By bootstrapping with replacement columns from the expression matrix, new realizations of the whole experiment can be generated. Consider for example the data matrix has 10 columns, a new realization of the experiment would consist a random sample of these 10 columns, e.g. columns {8, 3, 4, 6, 3, 4, 1, 1, 8, 9}. From these realizations, new lists of it p values and log fold changes can be calculated. We applied this approach 10 times on the expression matrix from R1 and 10 times on the expression matrix from R2.

### Bootstrapping sequencing reads from FASTQ-files (FB approach)

A FASTQ-file is typically generated as a result of a high-throughput-sequencing run and lists the sequences (called reads) of millions of DNA or RNA molecules, together with quality keys for the detection of each single nucleobase. To generate an artificial technical replicate, we draw $\pi \cdot k$ reads from the FASTQ-file with replacement, where *k* denotes the number of reads in the original file and $\pi$ is a percentage. Drawing reads with replacement means that a read from the original FASTQ-file can be selected multiple times for the bootstrap FASTQ-file. Here, we decided first to set $\pi =100\%$, so that the artificial replicate has as many reads as the original file. For computational efficacy, one can decide to use values of $\pi <100\%$, and we will also study and discuss this issue later. The reads from the new FASTQ-file are mapped again versus the reference genome to obtain read counts per gene. Then, new lists of it p values and log fold changes can be calculated. As with the CB approach, we run the FB approach 10 times, once from the FASTQ-files of R1 and once from the FASTQ-files of R2.

Due to the generation of additional FASTQ-files, this approach requires a higher amount of storage. Consider, one original FASTQ-file has a size of 5 GByte, and one wants to generate 10 artificial replicates using the FB approach, 50 GByte of additional storage would be necessary temporarily. Therefore, our strategy is to first generate *n* FASTQ-files with subsequent mapping and counting. After the differential analysis has been performed, artificially generated FASTQ-files are removed before doing the next run.

### Mixing columns of the expression matrix (MO approach)

Mixing observations is a typical technique of data augmentation regularly used to obtain more accurate machine learning models. To mix observations has particularly a long tradition in image classification. Here, we use a weighted mean of the columns of the following form to generate a new artificial replicates: $x_{new}=\sum _{i=1}^{n}w_ix_i$, where $x_i$ is the *i*th column of the expression matrix, i.e. a vector of length *d*, and $w_i$ are weights such that $\sum w_i=1$. In each replication of the experiment, we generate *n* new samples by this approach and bind them together as a new $(d \times n)$ expression matrix. As with the other two approaches, we replicate this approach 10 times, once based on the expression matrix of R1 and one based on the expression matrix of R2. Finally, the lists of it p values and log fold changes can be calculated.

### RNA-seq experiment on a Batai virus with technical replicates

In order to demonstrate the characteristics of each approach, we performed a small RNA-seq experiment with 4 independent control samples of the immortalized mouse dendritic cell lines DC2.4^18^ and 5 independent samples infected with *Batai orthobunavirus* (BATV)^19^. Cells used for infections as well as controls derived from the same batch were seeded in separate T25 ccm$^2$ tissue culture flasks (Sarstedt AG & Co., Nümbrecht, Germany) at the same density. Each infection was carried out with the same aliquot of BATV and RNA isolations were carried out 24 h post infection using QIAzolTM Lysis Reagent (Qiagen, Hilden, Germany). Each sample underwent library preparation using TruSeq Stranded mRNA library preparation kit (Illumina, San Diego, USA) and was run two times as technical replicate on an Illumina NextSeq 500, i.e. 18 samples were sequenced in total. The 18 samples were randomized to three NextSeq v2.5 Mid Output Kits to avoid additional technical batch effects. Raw data is provided in the NCBI Sequence Read Archive (please see ‘Availability of data and material’ statement), results of FastQC quality control are provided as supplementary material.

## Results

As with the data sets, we also denote the analytical results from the first sequencing run of the nine samples (5 infected versus 4 uninfected) by R1, and those from the second sequencing run of the nine samples by R2. It should be remarked that R1 and R2 were run simultaneously without a time gap. In the following, we call these also the true technical replicates, in contrast to the artificial replicates.

With each of the three strategies for generating artificial replicates, we produced 10 additional series of it p values and log2 fold changes, denoted in the following by FB1 to FB10 for the FASTQ-boostrap approach, by CB1 to CB10 for the column bootstrap approach, and by MO1 to MO10 for the mixed observations approach. The series of results also include those from the GO term enrichment analyses.

### Variability between the results from true technical replicates R1 and R2

**Figure 2:**
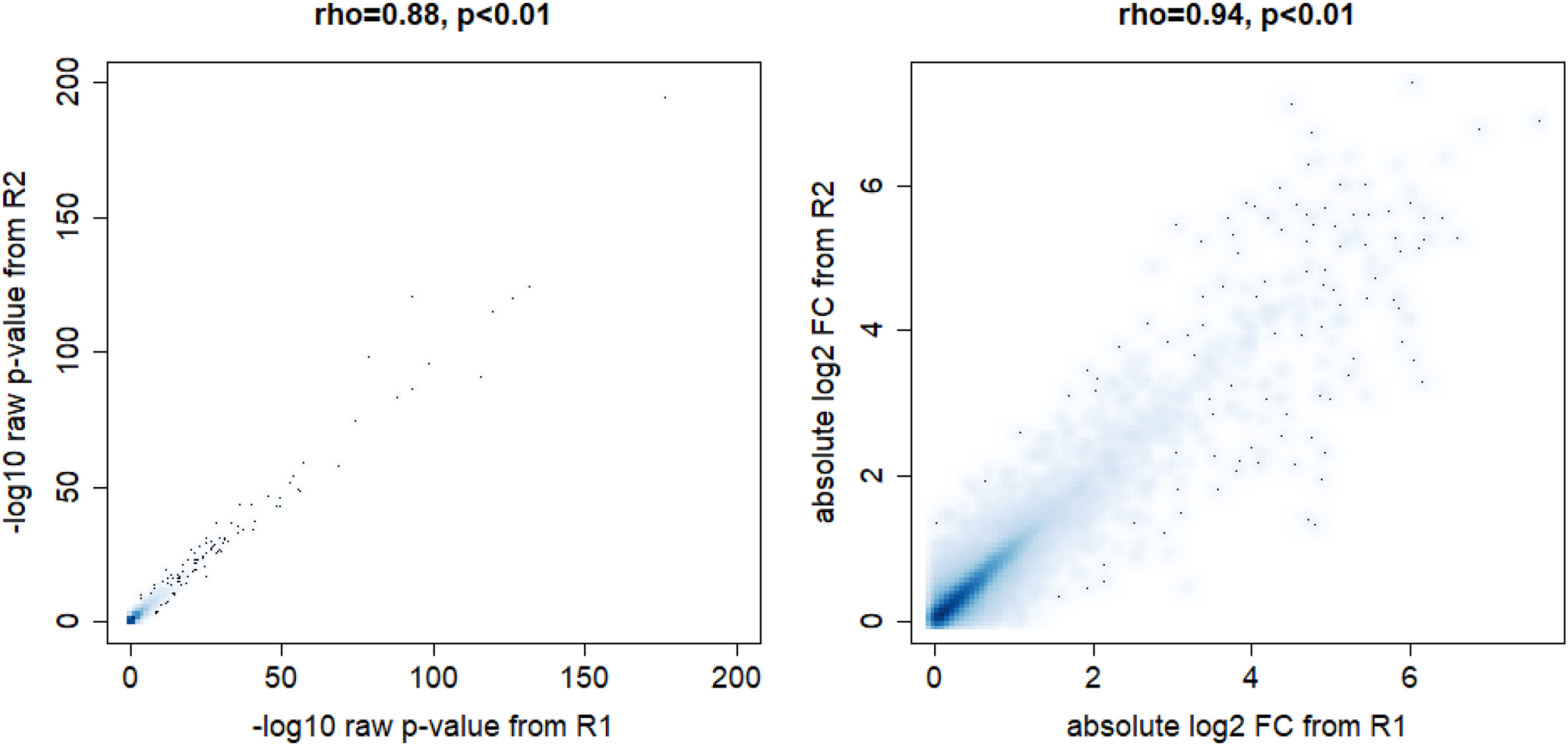
Smooth scatterplots of raw, gene-wise it p values generated from true experimental replicates R1 and R2 (left), and of absolute log2 fold changes (right). Spearman correlations between R1 and R2 are high, showing in principle a similar ranking of genes between technical replicates in an RNA-seq experiment. However, the plots also show a high number of genes which diviate stronger from the bisecting line, i.e. genes which would be selected by one experimental replicate but not by the other.

To assess the variability between the results from the two real technical replicates, R1 and R2, we compared the raw gene-wise *p* values as well as the absolute log2 fold changes of all genes after differential gene expression analysis (Fig. 2). We took the absolute log2 fold changes, because gene ranking would be based (besides on it p values) on the size of the fold changes independent of their sign, i.e independent of up- or down-regulation. As a measure of comparison, we used Spearman’s rank correlation coefficient $\rho$. In general, the results from both sequencing runs show significantly high rank correlation of gene-wise it p values and absolute log2 fold changes ($\rho =0.88$, $p< 0.01$ and $\rho =0.94$, $p< 0.01$, respectively). However, we can observe genes that deviate strongly from the bisecting line in both plots, indicating that a technical replication of the experiment can yield varying results. In extreme cases, this deviation can mean that some genes that have a significant it p value and a high fold change under R1 would not be selected as differentially expressed when the sample are sequenced a second time as technical replicate, and vice versa. Thus, even with a high correlation, biological conclusions drawn from the two replicates can differ.

### Similarity of replicates *p* values and log fold changes

As specified above, 10 new lists of it p values and log2 fold changes were produced per strategy. The similarity between these values in relation to those obtained from the two original replicates, R1 and R2, were compared in hierarchical cluster trees. We calculated the cluster trees where similarity between it p values was again measured using Spearman’s rank correlation coefficient. To be more precise, we used $1-\rho$ as a distance measure. In order to obtain compact clusters, we used the Ward method for clustering. When doing the comparison between results generated from real and artificial replicates, one should keep in mind that results from artificial replicates generated from R1 should reflect the results from the true replicate R2, and vice versa.

**Figure 3:**
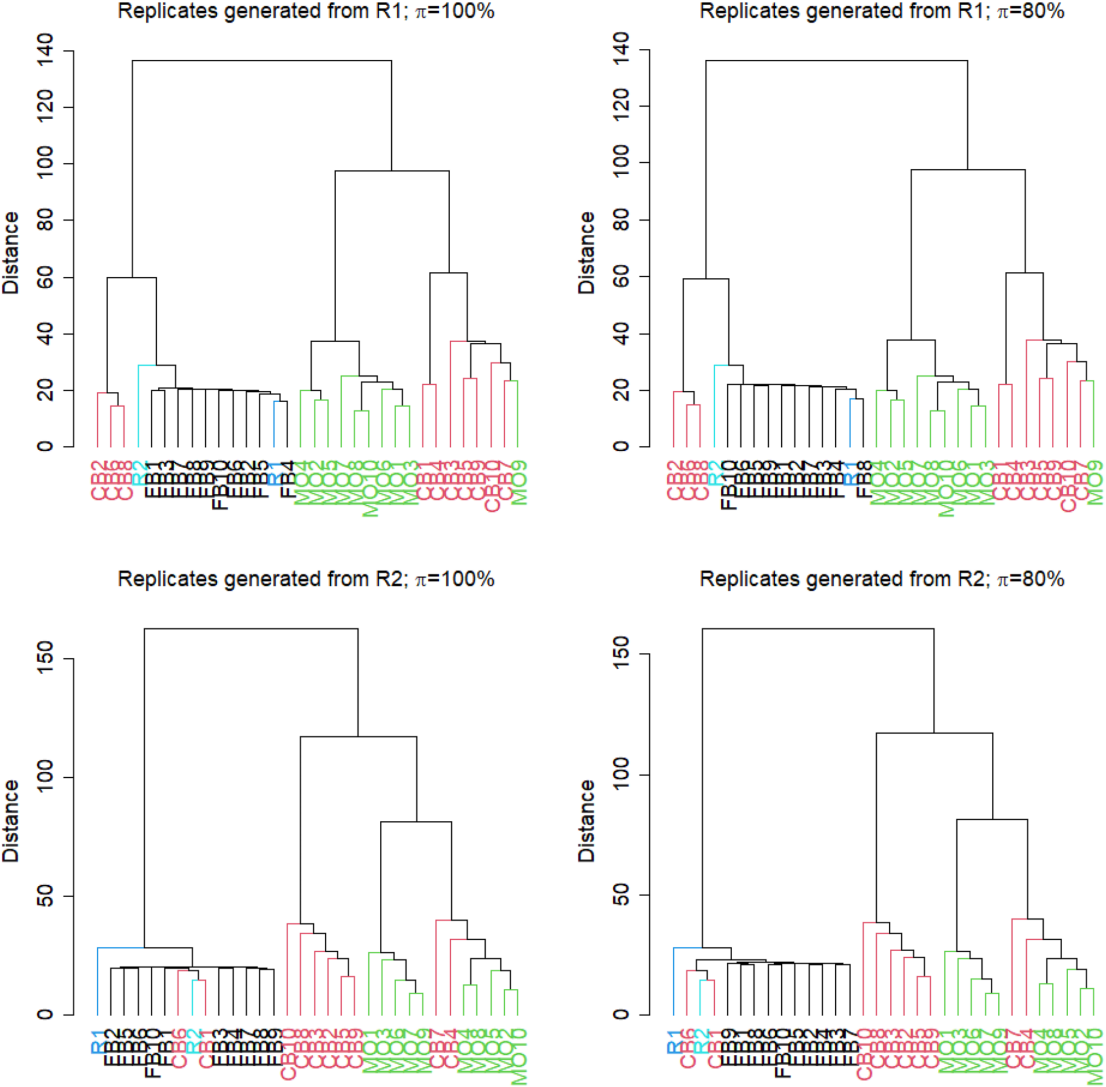
Cluster trees of it p value lists, where similarity of it p value lists was specified using Spearman’s correlation coefficient $\rho$. R1 and R2 denote the it p value lists obtained from the differential expression analysis of the original two replicates. FB1 to FB10 are the it p value lists from the approach of generating technical replicates by boostrapping reads from FASTQ-files. CB1 to CB10 denote the results after column boostrap and MO1 to MO10 denote the results obtained by the mixing observation approach. Cluster trees were generated either when artificial replicates were generated from R1 (top) or from R2 (bottom). Furthermore, FB replicates were either sampled with $\pi =100\%$ or $\pi =80\%$ of reads from the original FASTQ-files.

Figure 3 shows the relation of it p value lists obtained from the original two replicates and from the different approaches of building artificial replicates. Results are shown, when artificial replicates were either generated on the basis of data from R1 (Fig. 3, top) or from R2 (Fig. 3, bottom). Furthermore, results for the FB approach are either based on values of $\pi$=100% or $\pi$=80%. As detailed above, $\pi$ denotes the percentage of reads sampled relative to the original amount of reads in a FASTQ-file.

When generating FB results from R1, one can observe that the distance between results generated by the FB approach are closer to the results of the true experimental replicates R1 and R2 than results from the CB and MO approaches. When generating artificial replicates from R2 there are two results from the CB method that are as close to R1 and R2 as the FB results. Independent of generating results from R1 and R2, the results obtained from the MO approach and the majority from the CB approach clustered as outgroups. There is nearly identical clustering when changing the value of $\pi$. However, in regard of the desired aim, FB results are always too close to the original replicate from which artificial replicates were generated.

**Figure 4:**
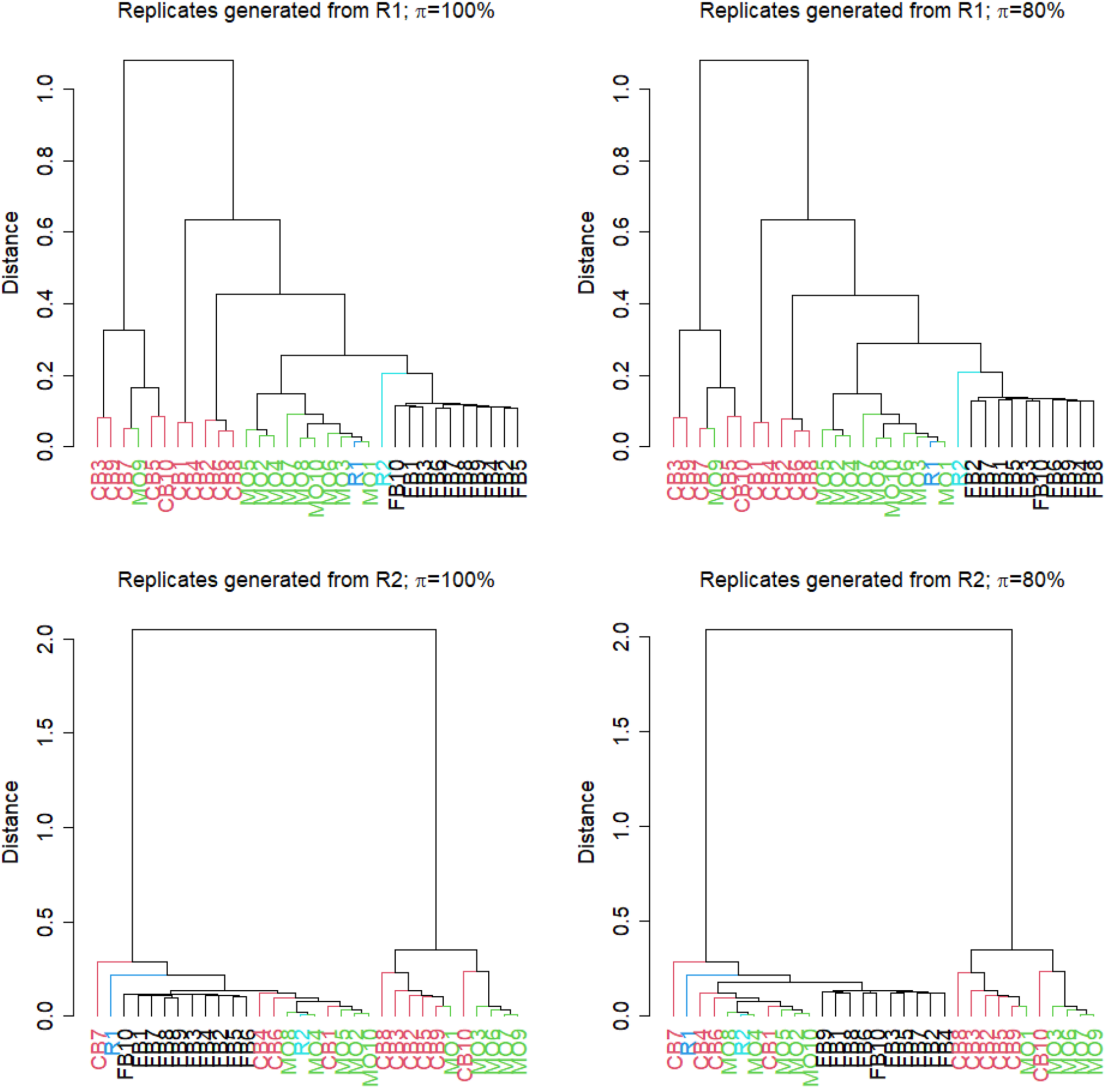
Cluster trees of log2 fold changes, where similarity of absolute log2 fold changes lists was specified using Spearman’s correlation coefficient $\rho$. R1 and R2 denote the log2 fold changes obtained from the differential expression analysis of the original two replicates. FB1 to FB10 are the log2 fold changes from the approach of generating technical replicates by boostrapping reads from FASTQ-files. CB1 to CB10 denote the results after column boostrap and MO1 to MO10 denote the results obtained by the mixing observation approach. Cluster trees were generated either when artificial replicates were generated from R1 (top) or from R2 (bottom). Furthermore, FB replicates were either sampled with $\pi =100\%$ or $\pi =80\%$ of reads from the original FASTQ-files.

When comparing the log2 fold changes we observe a slight change in the cluster trees that are generated the same way as those for the it p value lists (Fig. 4). When artificial results were generated from R1, one can observe that the results obtained from the MO approach are closest to R1, but results obtained from the FB method also cluster close to R1 and very close to R2. When results were generated on the basis of R2, the results from MO and CB approaches are more divided within the cluster trees. Half of the results obtained by the MO approach are close to R2 and the other half is clustered together with the majority of the CB results as an outgroup. In general, the observations for log2 fold changes are more in favour of the FB approach than could be observed for the analysis of it p values. In fact, results based on artificial replicates generated from R1 cluster closer to the results from R2, and vice versa.

Although there are still samples that managed to be clustered close to the two technical replicates the FB approach seems to be more consistent in both cases, when artificial results are generated from R1 or R2. Also, in both comparisons the results obtained from the FB approach are always clustered together and are close to both technical replicates regardless of the value $\pi$.

Besides comparing the results from differential expression analysis in form of it p values and log2 fold changes, we also compared directly the similarity between original gene expression data and that from the artificial replicates. Cluster trees, separately for treatment and control data, is provided as Supplementary Figures S5 and S6. The overall picture in these plots is not as clear as in the cluster trees of it p values and log2 fold changes. The FB and MO generated data cluster relatively close to R1 and R2 while several (but not all) CB data cluster further away from R1 and R2. In order to understand, whether the correlation between original data R1 and R2 with the artificial data depends on specific factors, we generated some exemplary scatter plots (Supplementary Figure S7). The overall shape was very similar in other replicates generated by the three approaches. It can be seen from the correlation plots that in general the distribution of highly expressed genes remains more stable with each of the three approaches, while there is less stability for low expressed genes. We could not identify another particular influence on the stability of gene expression values.

### Overlap of selected genes and GO terms

**Figure 5:**
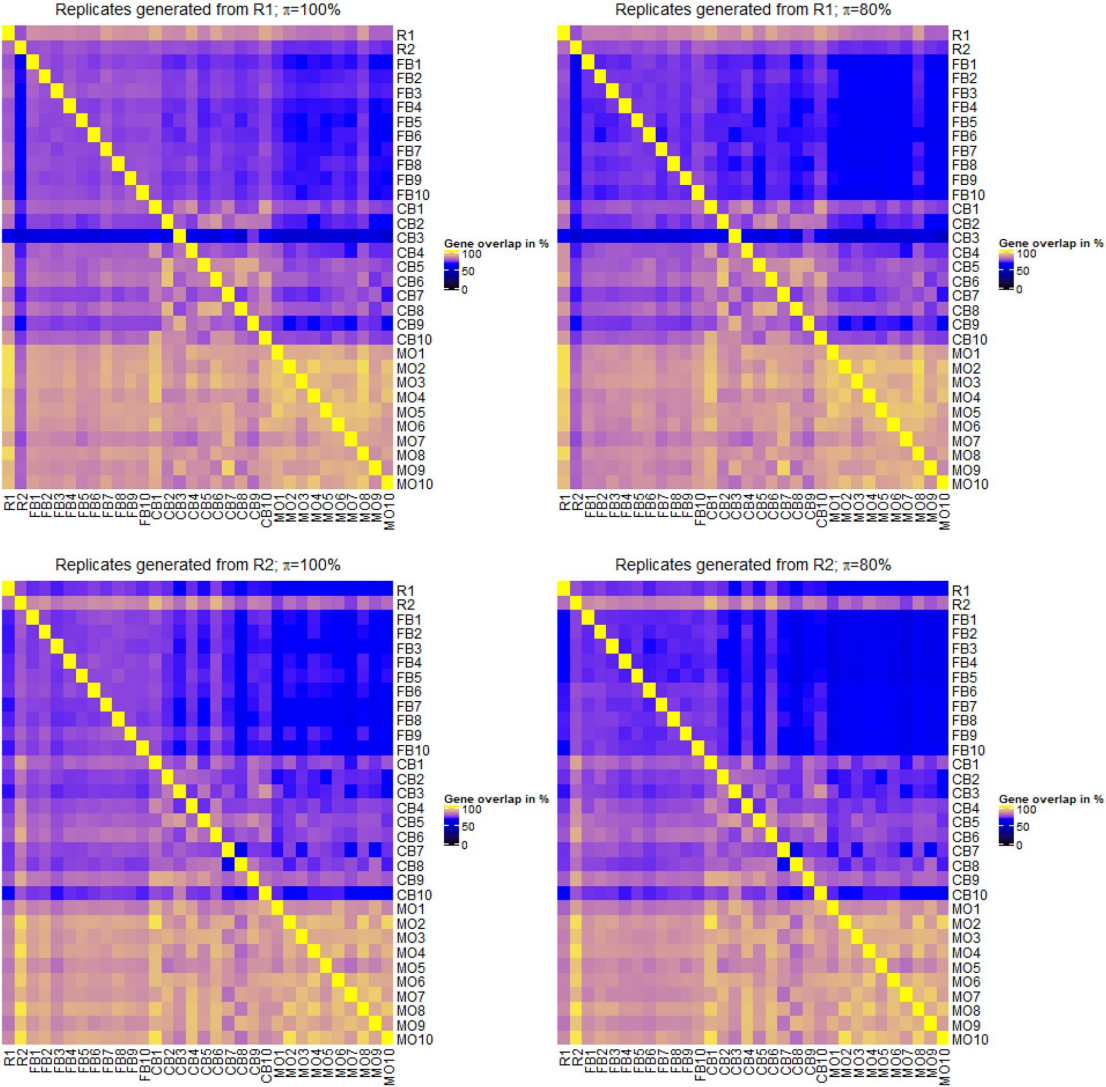
Heatmaps reflecting the overlap of selected differentially expressed genes from true experimental replicates R1 and R2 as well as from the artificial replicates with the three different approaches. Again, heatmaps are shown when artificial replicates were generated from R1 (top) or R2 (bottom) and with different values $\pi$ for the FB apprach. Overlap is given in percent of genes detected in a comparison analysis (column) with respect to an reference analysis (row). Exemplarily, the interpretation for the plot top left is as follows. Replicates were generated from the data of R1 with the purpose of obtaining similar results as from R2, which is shown in the second line. Here, FB and CB results show a stronger overlap with R2 than results from the MO strategy. Heatmaps were drawn using the R-package ‘ComplexHeatmap’ (version 2.6.2., www.bioconductor.org).

While the above cluster trees are based on results for all genes, we now focus only on the genes selected as differentially expressed and GO terms selected as significantly enriched. Heatmaps in Fig. 5 show the overlap in percentage of differentially expressed genes between each pair of true and artificially generated replicates. Again, one plot shows the results when artificial results were generated from R1 (top plot) and the other plot when samples were generated from R2 (bottom plot). Each data set is represented in one column and one row as well. Remark that the overlap matrices shown in the heatmaps are not symmetric. Each row represent a reference data set and the columns the data sets with which the set of selected genes in the reference data set is compared. Differentially expressed genes were selected using a threshold of 0.05 for FDR-adjusted it p values and a threshold of $+/-2$ for the log fold change.

When artificial samples are generated from R1 we wish to have a large overlap with the results from R2. Therefore, we first look at the second row of Fig. 5 (top left): genes selected from the FB data sets show a little bit more overlap with genes selected from R2 than genes selected from CB and MO data sets. The same results can be seen from the first row in Fig. 5 (bottom left), when artificial samples were generated from R2.

What we also see from the heatmaps is the correlation between the results of each individual method and between R1 and R2. From data sets R1 and R2, 595 and 565 genes are selected with the above defined thresholds, respectively, with an overlap of 447 genes. I.e., 447/595 = 75% of genes selected from R1 are also found from R2, and 447/565 = 79% of genes selected from R2 are also found from R1. The overlap of selected genes between the FB data sets ranges between 67 and 76%. Within CB and MO data sets the overlap ranges from 55 to 94%, and from 79 to 96%, respectively. Thus, there is much higher variability in the results generated from CB and MO data sets than in the results from FB data sets. Moreover, the size of overlaps between results from FB data sets is more similar to the overlap between results from R1 and R2, compared to the size of overlap between CB and MO samples.

The heatmaps also emphasise that results from MO replicates are more different from results from FB and CB replicates, which was already given in the cluster trees of Fig. 3. On the one hand, most genes selected by the MO replicates are also found by the other approaches (yellow parts in the areas at the bottom). On the other hand many genes selected by the FB and CB replicates are not found by the MO approach (blue areas at the top and center right of the heatmaps. While plots in Fig. 3 are based on all it p values, it appears that the distance of MO replicates to the other results is even more brought out after gene selection.

**Figure 6:**
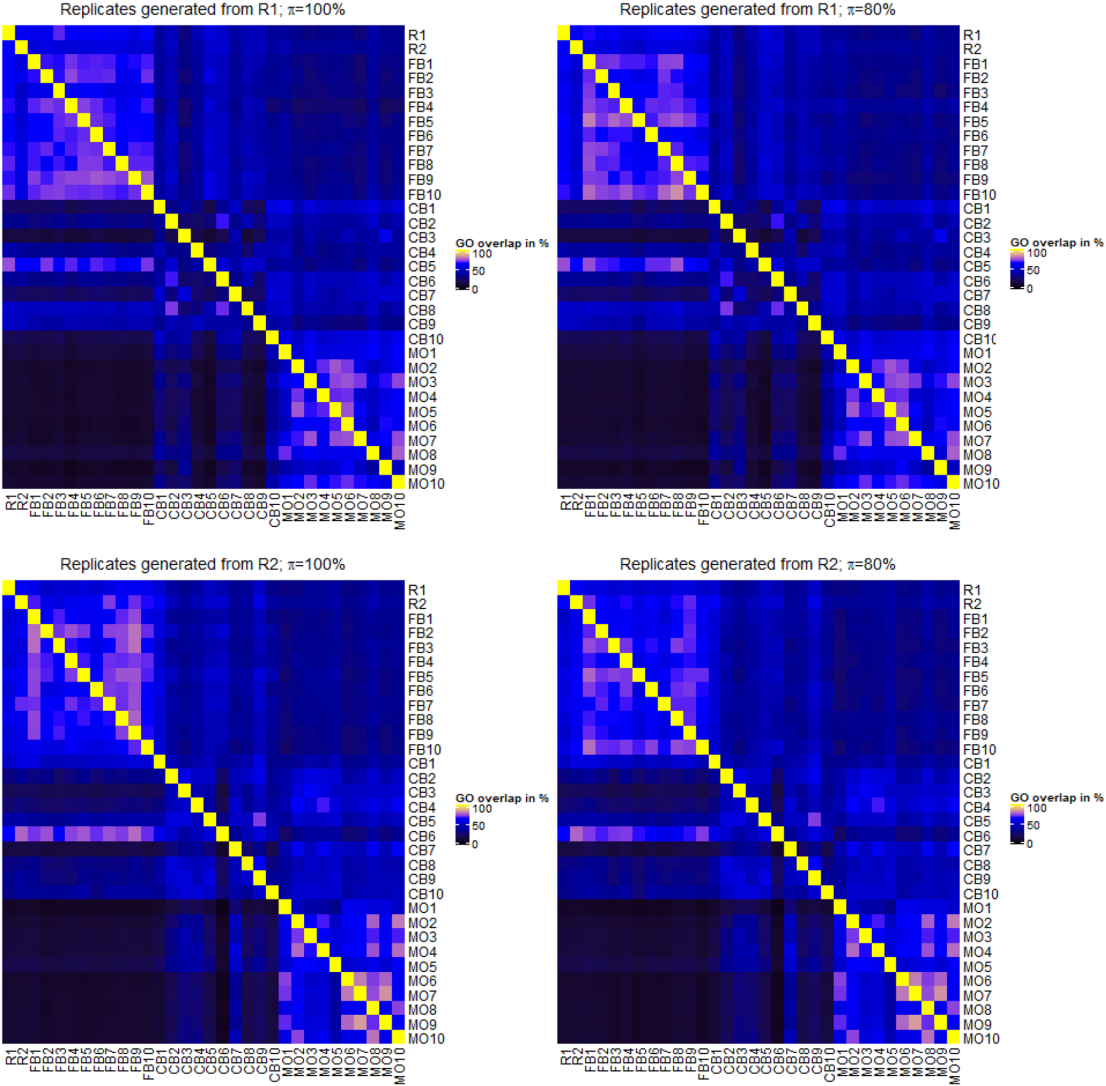
Heatmaps reflecting the overlap of selected enriched GO terms in the same way as the heatmaps of differentially expressed genes. Heatmaps were drawn using the R-package ‘ComplexHeatmap’ (version 2.6.2., www.bioconductor.org).

Since enrichment analysis of GO terms is based on the it p value lists obtained from the differential expression analysis, it is very likely that the performance of methods for artificial replicates is similar as described above. Indeed, one can observe a similar trend of results (Fig. 6). Overall, results produced by the FB approach have more overlaps with results from R1 and R2 ($>50\%$) in enriched GO terms compared to the overlap of results from the CB and MO approach with R1 and R2 ($<50\%$).

### Dispersion across data sets

**Figure 7:**
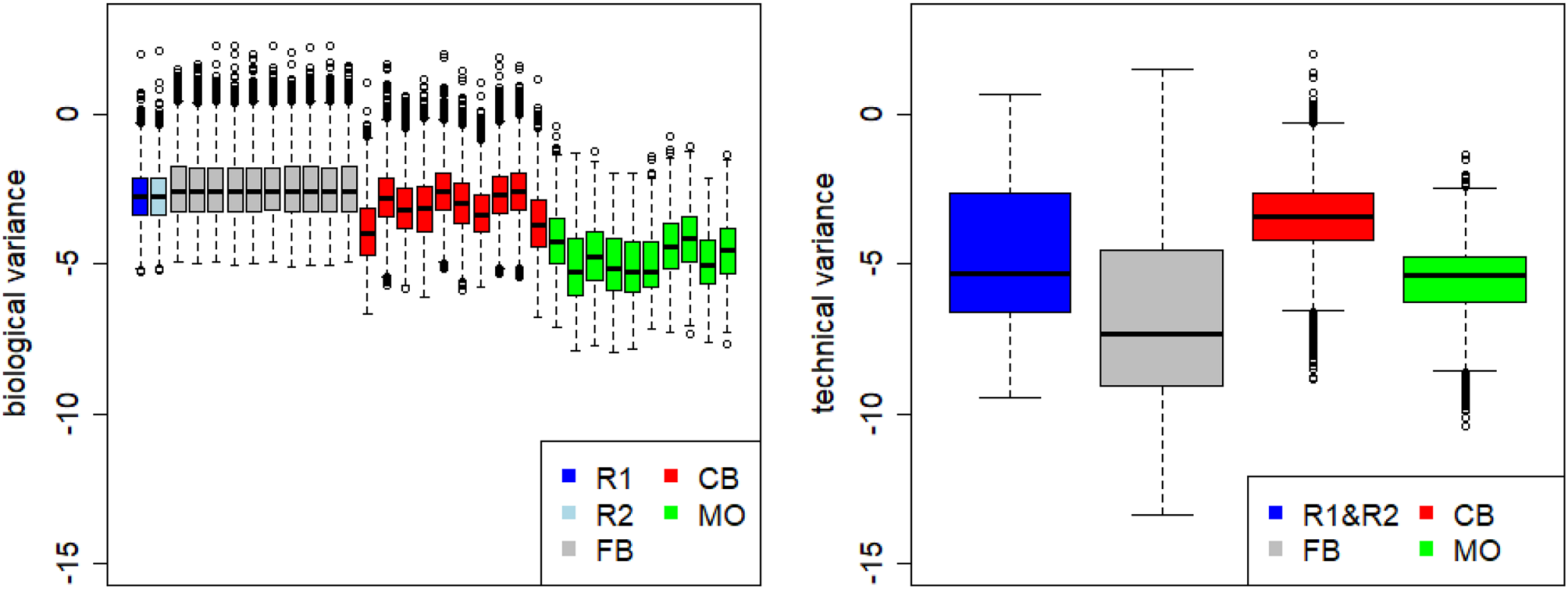
Left: distribution of gene-wise dispersions estimated in the real and artificial data sets. These dispersions reflect the biological variance of the nine samples. Right: distribution of gene-wise dispersions between true replicates R1 and R2, as well as between all FB, all CB and all MO samples. These distributions reflect the variance between each set of replicates.

As part of differential expression analysis, DESeq2 determines gene-wise dispersion estimates which can be used to determine biological or technical variance in different subsets of the data. For the following analysis, biological and technical variance were extracted for approx. 30 thousand genes to which the reads of the original FASTQ-files mapped. The distribution of gene-wise biological dispersion estimates obtained from data sets R1, R2, FB1–FB10, CB1–CB10, and MO1–MO10 are shown in Fig. 7 (left plot), where each boxplot represent approx. 30 thousand variance estimates. As can be seen, the distributions of biological variances determined in the artificial data sets FB1–FB10 are more similar to those of R1 and R2 than to those obtained in data sets CB1–CB10 and MO1–MO10. Additionally, the distributions in the 10 FB data sets are more homogenous, while the results from the CB and MO data sets show a high variability.

To determine the technical variance, dispersions were estimated in different subsets: R1 plus R2, all FB, all CB or all MO data sets, respectively. In this setting, not the experimental group factor (infected versus control) but the nine biological replicates where put as factor in the design matrix of DESeq2. Thus, the dispersion estimates reflect the technical variance between the replicates. The analysis yields that neither of the three strategies reflects the technical variance appropriate. The FB and the MO approach under-estimate the variance for most genes, while dispersions estimated by the CB approach are mostly larger than in the true replicates (Fig. 7, right plot).

### Tuning variability of FASTQ-bootstrap replicates

We evaluated the FB approach by tuning the amount $\pi$ of sampled reads from the original FASTQ-file. Changing $\pi$ from 100 to 80% seems to have no noticeable impact on the results represented by detected differential expressed genes or identified enriched GO terms (Figs. 5 and 6) and by pair wise comparisons of it p value lists and log2 fold change lists (Figs. 3 and 4). We further reduced the size of $\pi$ to values of 75% and 50%, respectively, and observed that the it p values and log2 fold changes do not change strongly. A value of $\pi$=75% still produces results close to R1 and R2 in comparison to the results obtained from the CB and MO approaches. However, when reducing $\pi$ to a value of 50% the amount of detected differential expressed genes decreases strongly. Compared to $\pi$=100%, where approximately 80% of genes represented in R1 are also detected by the FB approach, a $\pi$ of 50% results in the detection of less than 50% of those genes. Furthermore, less than 30% of differentially expressed genes detected with a $\pi$ of 50% are also detected in R1. Heatmaps and cluster trees for results with $\pi =75\%$ and $\pi =50\%$ are provided as Supplementary Figures S1–S4.

### Application of FASTQ-bootstaps for the analysis of the BATV experiment

Based on the above findings that the FB approach produces artificial technical replicates that behave similar as true replicates, we want to demonstrate here, how this approach can be used to rate uncertainty in an RNA-seq experiment. In the RNA-seq experiment on the BATV, 628 genes were selected in the R1 replicate with an FDR-adjusted it p value $<5\%$ and an absolute log2 fold change $>2$. Only 346 of these genes were again selected in 100% of 10 replicates of the whole experiment generated by the FB approach, additional 77 genes were selected in 90% of the FB replicates. One of the 628 genes was not selected by either of the FB replicates. Furthermore, 1059 genes were additionally found in the FB runs but not in R1. However, 934 of these 1059 were detected in less than 50% of the FB runs, thus these additional findings would not be too critical. The complete comparison between numbers of selected genes in R1 and the 10 FB runs are given in Table 1.

**Table 1:** Comparison of genes selected as differentially expressed in true technical replicate R1 (TRUE=gene was selected by adjusted it p value and log2 Fold Change; FALSE=gene was not selected) and in 0, 1, ..., 10 runs with artificially generated technical replicates by the FB approach. Of 628 genes selected from the R1 data, only 346 genes were selected in all 10 runs of the FB approach.

		**FB approach**										
		0	1	2	3	4	5	6	7	8	9	10
**R1**	FALSE	–	524	210	131	78	57	39	21	7	1	0
	TRUE	1	2	4	9	19	31	34	51	54	77	346

**Figure 8:**
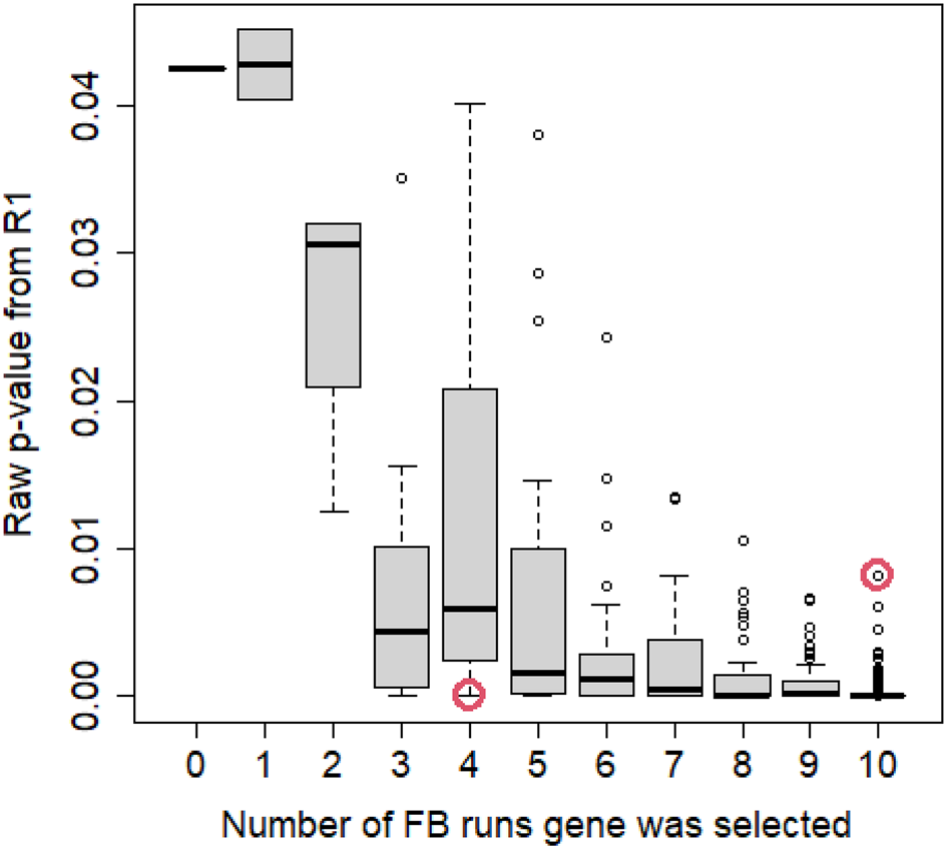
Raw it p values of 628 genes selected as differentially expressed by in the R1 data set versus number of runs with artificially replicates generated by the FB approach.

Figure 8 shows the raw it p values of the 628 selected genes from R1 and the number of runs a gene was selected in the replicates generates by the FB approach. Typically, genes are ranked by their raw it p value in a differentially expression analysis (adjusted it p values can contain bindings leading to a less accurate ranking, and fold changes don’t contain information about variance and significance.) The two genes indicated by red circles in this figure represent extrem scenarios. The gene that was selected in only 4 FB runs would obtain a higher rank than the gene selected in 10 FB runs, however, the bootstrap approach points at a higher uncertainty for the former one.

## Discussion

Reproducibility of scientific results has been discussed widely in the recent years, also including its perception as a ‘crisis’^20^. In general, reproducibility means the ability to reproduce another scientist’s results, or eventually to reproduce own results. Primarily, this can be translated to the ability of performing an experiment that leads to the same scientific conclusions as a prior experiment. Other aspects of reproducibility include reproducible analysis, i.e. the availability of data and analysis code, and reporting guidelines^21,22^.

Although RNA-seq seems to have a high level of reproducibility, results still can change when technically replicating the experiment. In consequence, this can change the biological interpretation when selected genes or GO terms fall out or come into the list. For example, falsely identified induced or repressed pathways may lead to costly follow-up experiments, which will not lead to informative data or in the worst case wrong therapeutic approaches. However, it is state of the art to validate candidate genes and pathways by an independent method such as qRT-PCR or Proteomics approaches before planning therapeutic interventions or follow-up animal experiments. This effect of altered interpretation based on different replicates data was also seen in the technical replication of our case study.

Understanding the technical reproducibility of an RNA-seq experiment is also important since several studies have demonstrated that the way of analysis and of results reporting can additionally affect the reproducibility of results^22,23^. Therefore, we have studied the usability of artificially generated replicates for measuring the uncertainty of selected molecular features. While the CB and MO approach may also be helpful to study the range of possible results when replicating an RNAs-seq experiment, the FB approach appears to be better suited since these data and results obtained from their analysis are closer to real technical replicates. The proposed methods are cheap to perform in contrast to true replicates, however, additional storage space per analysis must be allowed for, in particular for the FB approach.

Analysis of estimated dispersions has shown that the FB approach maintains the distribution of biological variances very well, but under-estimates the technical variance. The two other approaches do neither maintain the biological variance observed in the true data nor reflect the technical variance seen in the true replicates. Although the other results for the FB method are promising, an under-estimation of the technical variance can lead to an too optimistic view on the reproducibility of an experiment.

For specific questions concerning direct effects of the sequencing machine, such as lane effects of the flow cell, artificially generated replicates are, however, not helpful. Furthermore, we have not yet studied how many artificial replicates of an experiment would be necessary. Here, we used 10 instances for each of the three strategies and think that this number will be sufficient in most scenarios to judge whether selected genes and GO terms from the true samples would be selected if the experiment was replicated.

It also appears that artificially replicates generated by the studies approachs don’t have an effect on the power to detect differentially expressed genes. The improvement of power when using technical replicates on different lanes of the flow cell was mentioned by Marioni et al.^4^, but we did not explicitly investigate this effect in our analysis. Marioni et al. argue that the number genes whose expression can be assessed increases with the number of replicates. Consequently, there would also be an increased power to detect differentially expressed genes. When using the FB approach to generate technical replicates artificially reads are drawn always from the same original set of reads. For the MO and CB approach, it is also very clear that no additional genes will be involved. Therefore, the FB approach will not lead to additional genes whose expression can be assessed.

The approach of bootstrapping from the FASTQ-files has been demonstrated useful in metagenomics based on high-throughput sequencing, and may also be applicable to many other applications of high-throughput sequencing that produce FASTQ-files such as variant analysis^24^, single-cell RNA-seq^25^ or methylation analysis^26^. In general, the possibility of using simulated technical replicates allows scientists to commit available resources to including more biologically independent replicates, which is more relevant to the reproducibility than technical replicates^27^. Although we have not studied the possibility of using the FB approach for the purpose of data augmentation in machine learning, this could be worth a question for further research. The observations from the direct data comparison (Supplementary Figures S5 and S6 suggest that the FB and MO approaches may be useful for data augmentation in the context of machine learning. For the purpose of judging the uncertainty of differential expression and enrichment analysis, we think that our findings from Figs. 3 and 4 are more informative.

## Conclusions

Bootstrapping sequencing reads from FASTQ-files is a helpful approach to generate artificial replicates in RNA-seq experiments. It has been shown in our example experiment that data generated by the FB approach show a similar behavor as true experimental replicates. In contrast, replicates generated by the CB and MO approaches show a higher variablity among each other than the variability among true replicates. While with the FB approach, information of each original sample is preserved in the artificial replicates, information of individual samples can be lost when using column bootstrap (CB) or weighted mixed observations (MO). The FB appproach can be used to better judge uncertainty and reproducibility of genes selected as differentially expressed between two groups of samples. In particular, results from differential expression analysis on the FB generated replicates can be used to judge the robustness of ranking lists obtained from the true data. In addition, the FB approach can be used for the same purpose in subsequent gene set analysis such as gene-set enrichment analysis. One drawback of the FB approach is it’s high computational cost.

## Supplementary Information

Supplementary Information 1.

Supplementary Information 2.

Supplementary Information 3.

## Data Availability

The datasets generated and/or analysed during the current study are available in the NCBI Sequence Read Archive (SRA) repository, https://www.ncbi.nlm.nih.gov/sra/PRJNA764858.

## Abbreviations

BATV: Batai virus
CB: Column bootstrap
FB: FASTQ-bootstrap
GO: Gene ontology
MO: Mixed observations
R1, R2: Replicate 1, replicate 2
