# Supplementary material for: A comparison of strategies for generating artificial replicates in RNA-seq experiments
Source: Sci Rep. 2022 May 3;12:7170. doi: 10.1038/s41598-022-11302-9 (PMC9065086; doi:10.1038/s41598-022-11302-9)
Supplement: Supplementary file 1 — Supplementary Information 1. [file 41598_2022_11302_MOESM1_ESM.pptx]

## Slide 1
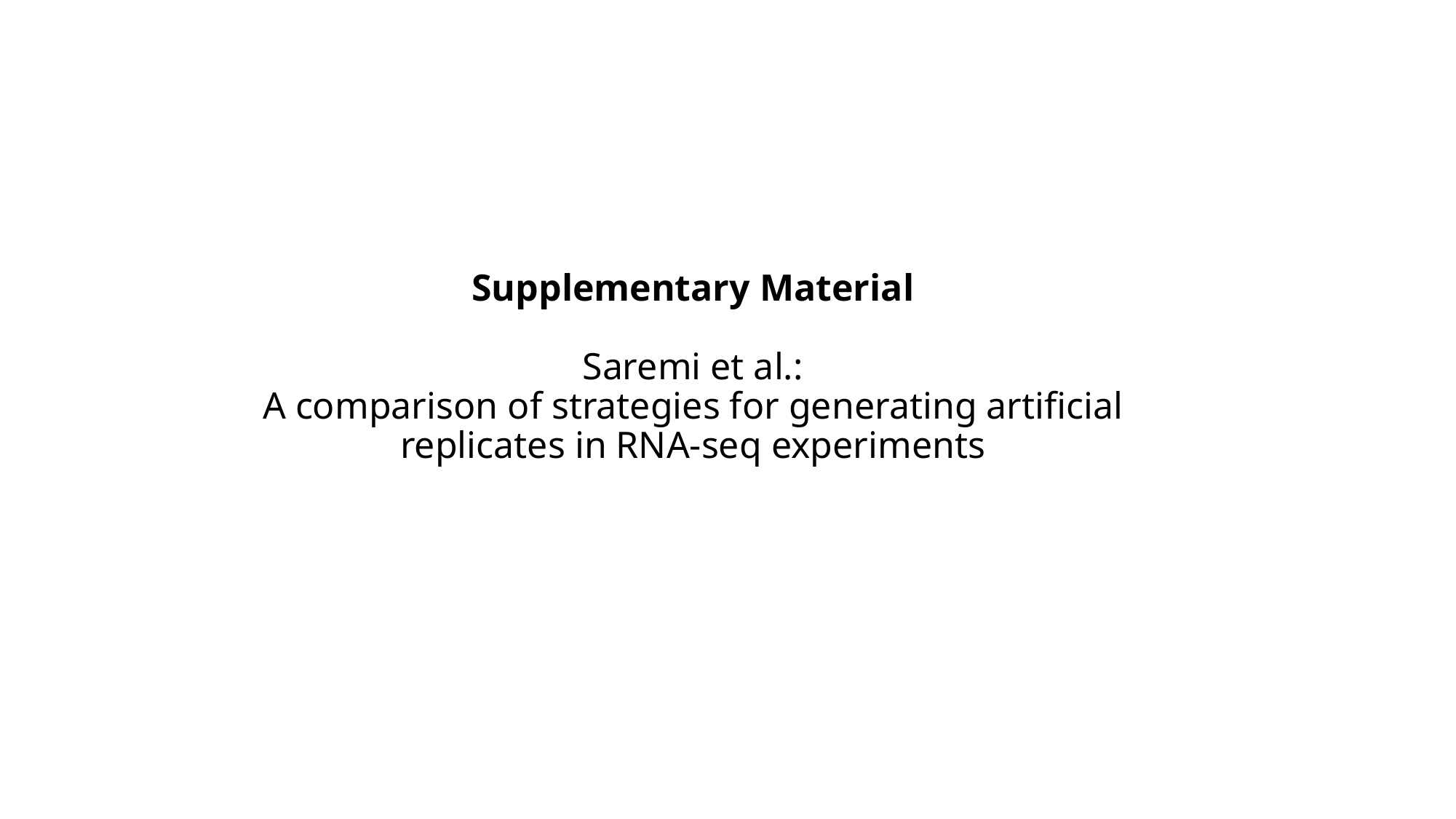

# Supplementary Material Saremi et al.: A comparison of strategies for generating artificial replicates in RNA-seq experiments

## Slide 2
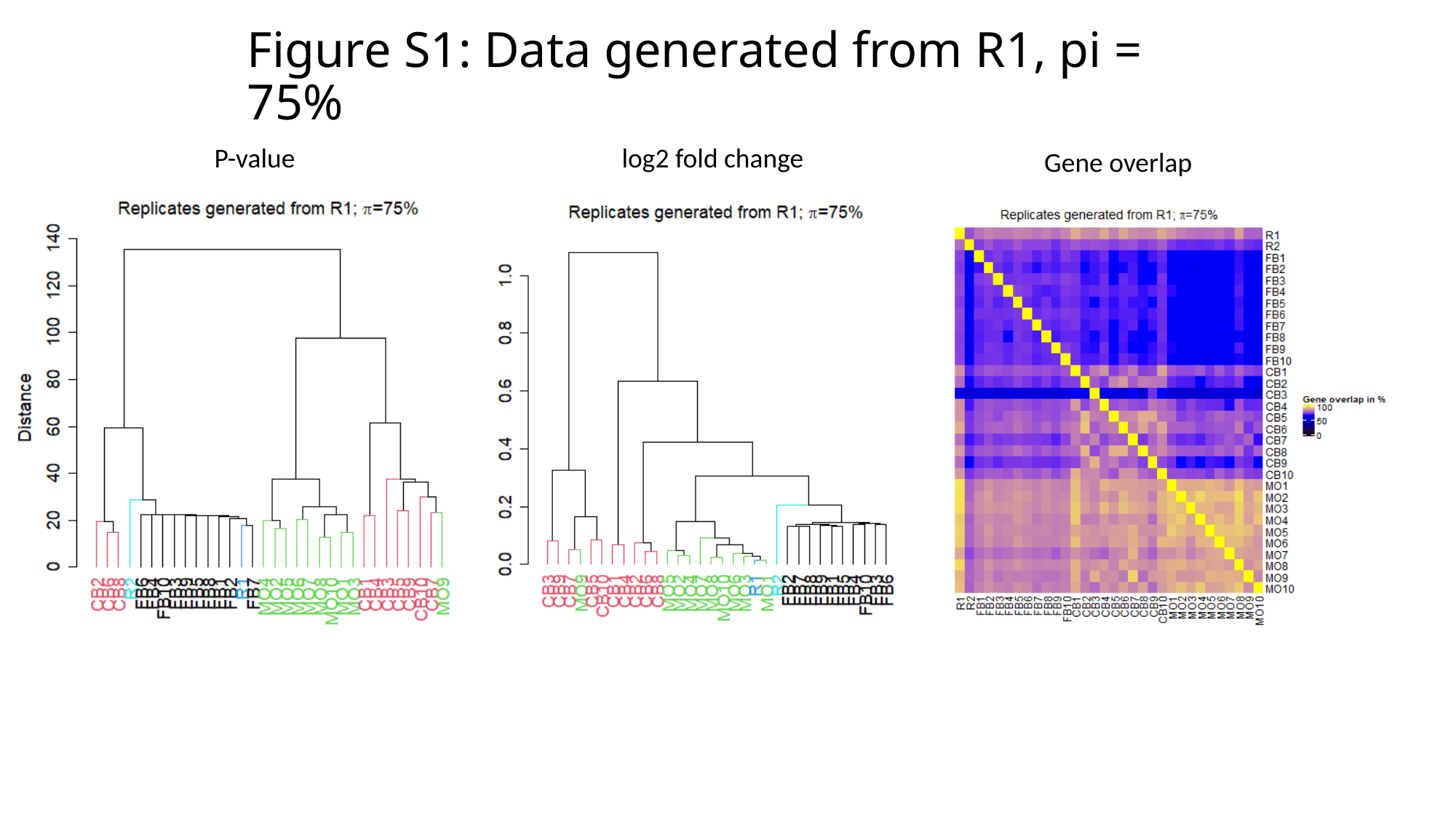

# Figure S1: Data generated from R1, pi = 75%
P-value
log2 fold change
Gene overlap

## Slide 3
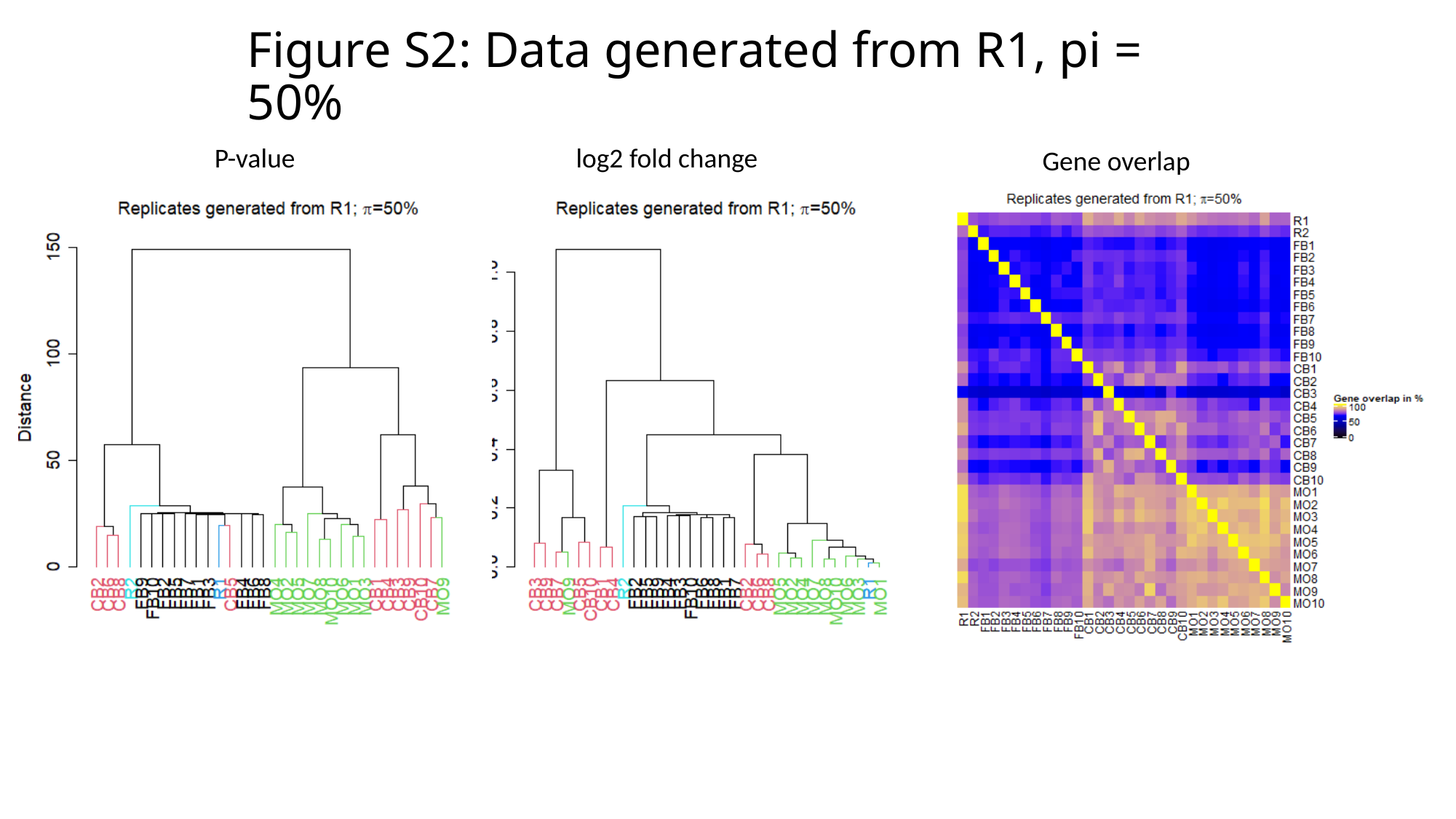

# Figure S2: Data generated from R1, pi = 50%
P-value
log2 fold change
Gene overlap

## Slide 4
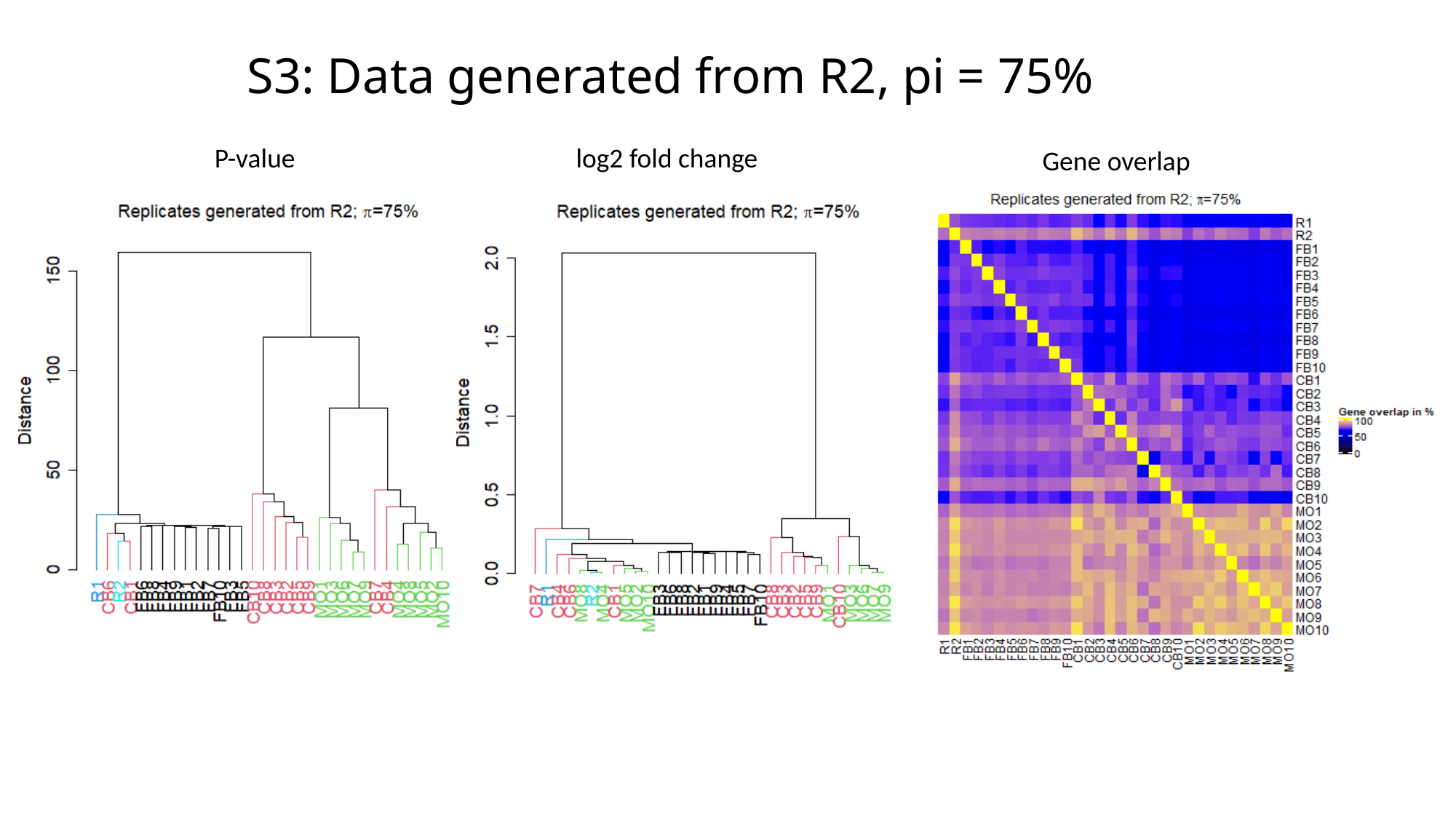

S3: Data generated from R2, pi = 75%
P-value
log2 fold change
Gene overlap

## Slide 5
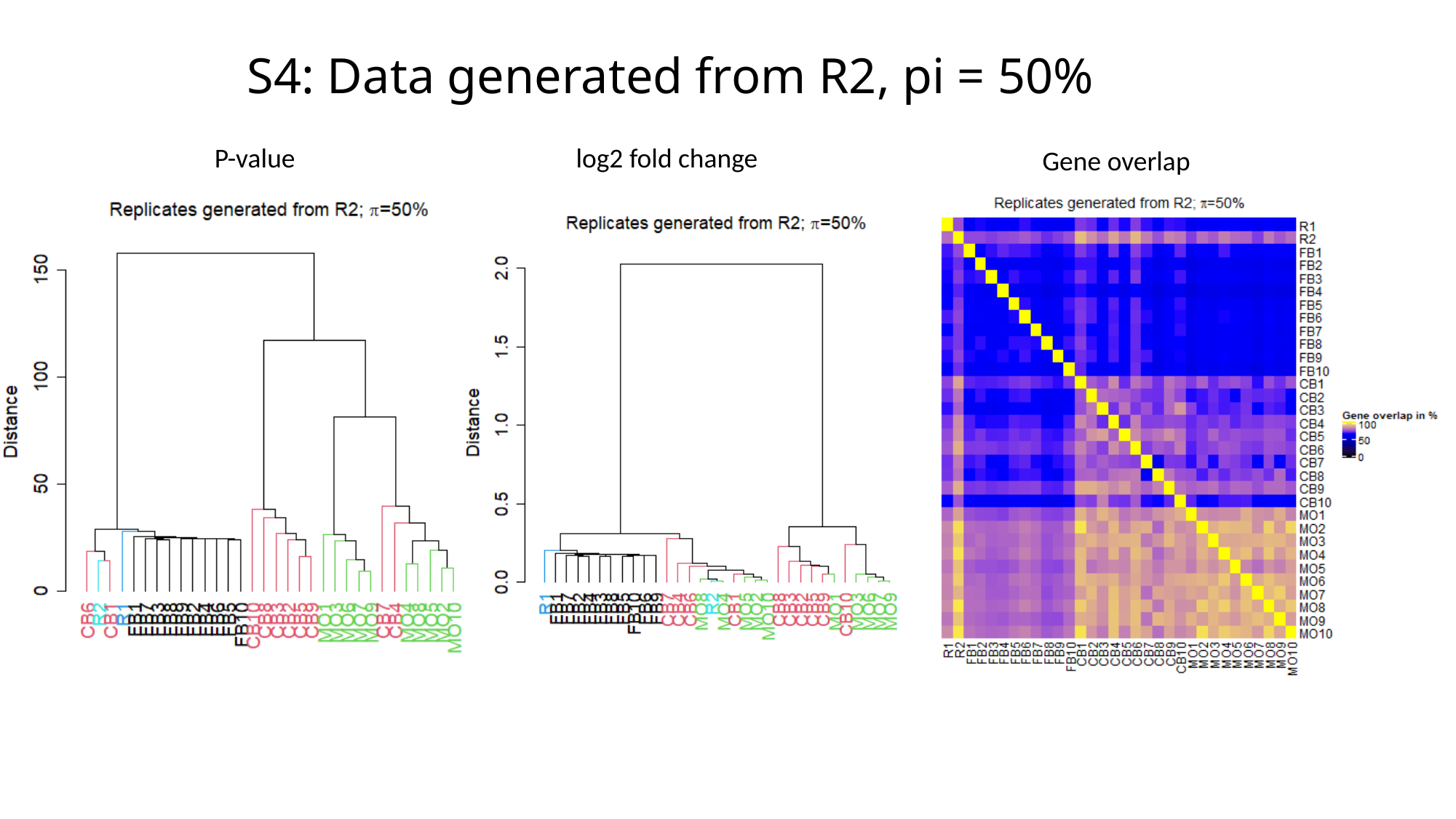

# S4: Data generated from R2, pi = 50%
P-value
log2 fold change
Gene overlap

## Slide 6
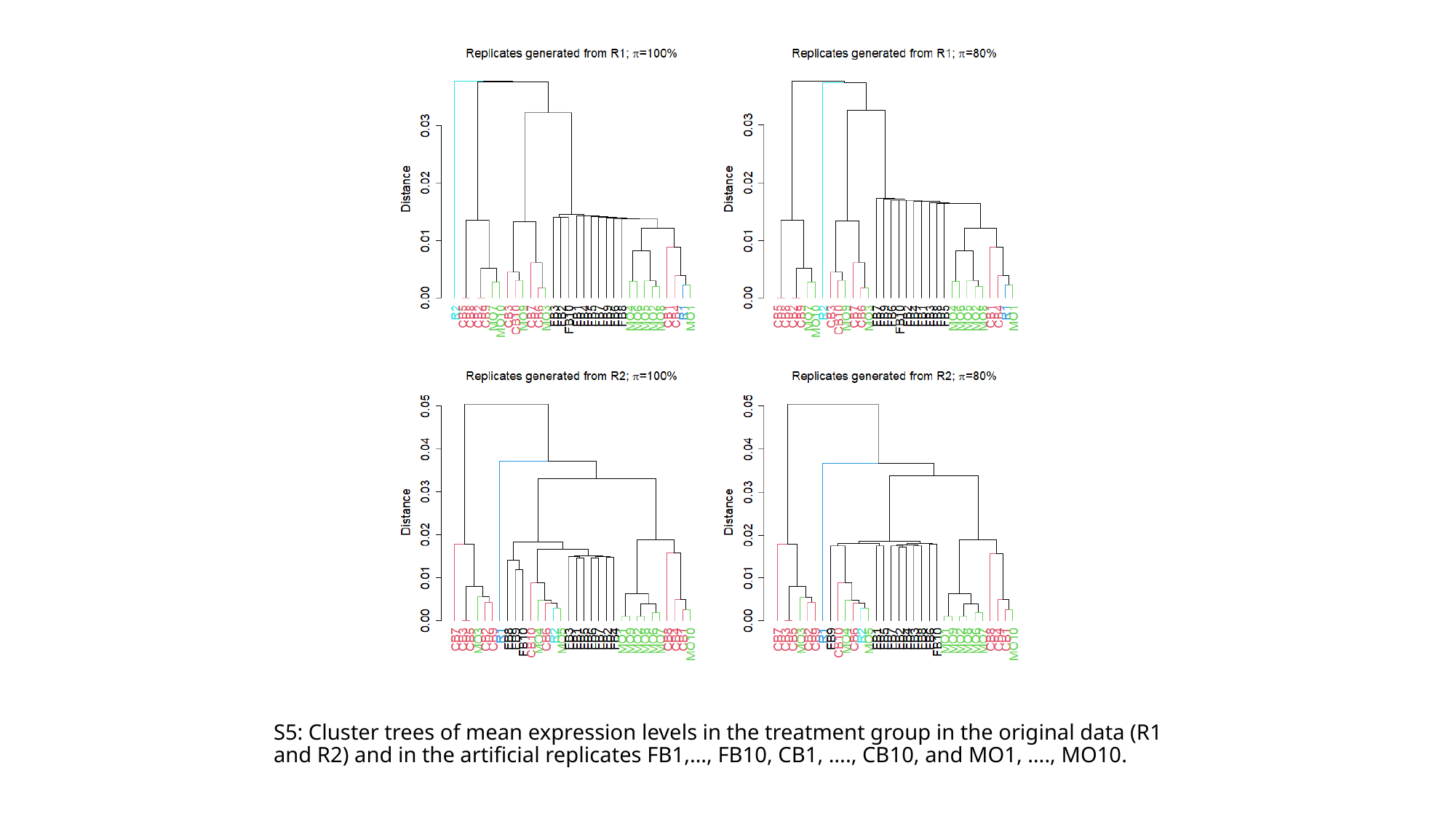

# S5: Cluster trees of mean expression levels in the treatment group in the original data (R1 and R2) and in the artificial replicates FB1,…, FB10, CB1, …., CB10, and MO1, …., MO10.

## Slide 7
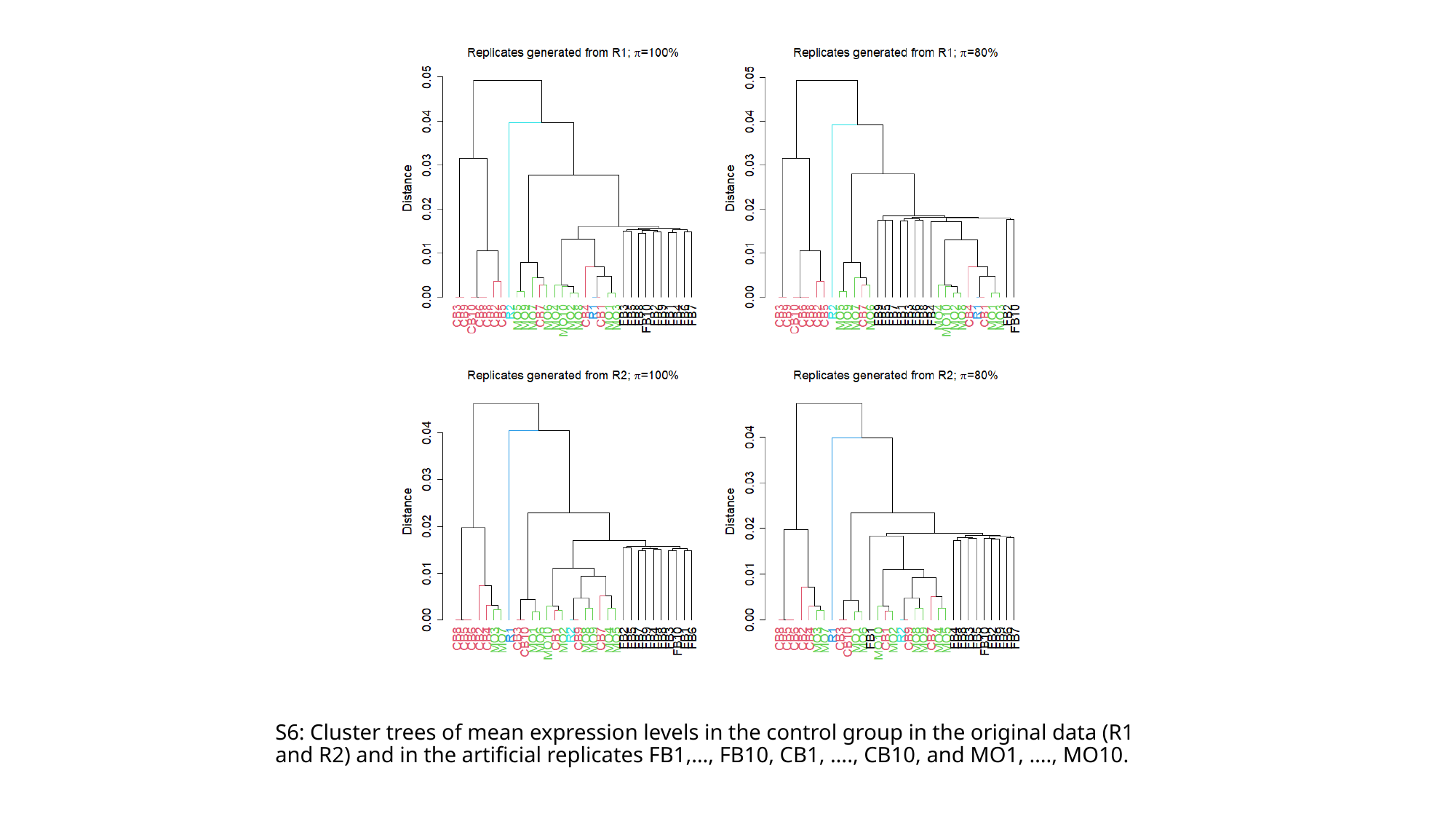

# S6: Cluster trees of mean expression levels in the control group in the original data (R1 and R2) and in the artificial replicates FB1,…, FB10, CB1, …., CB10, and MO1, …., MO10.

## Slide 8
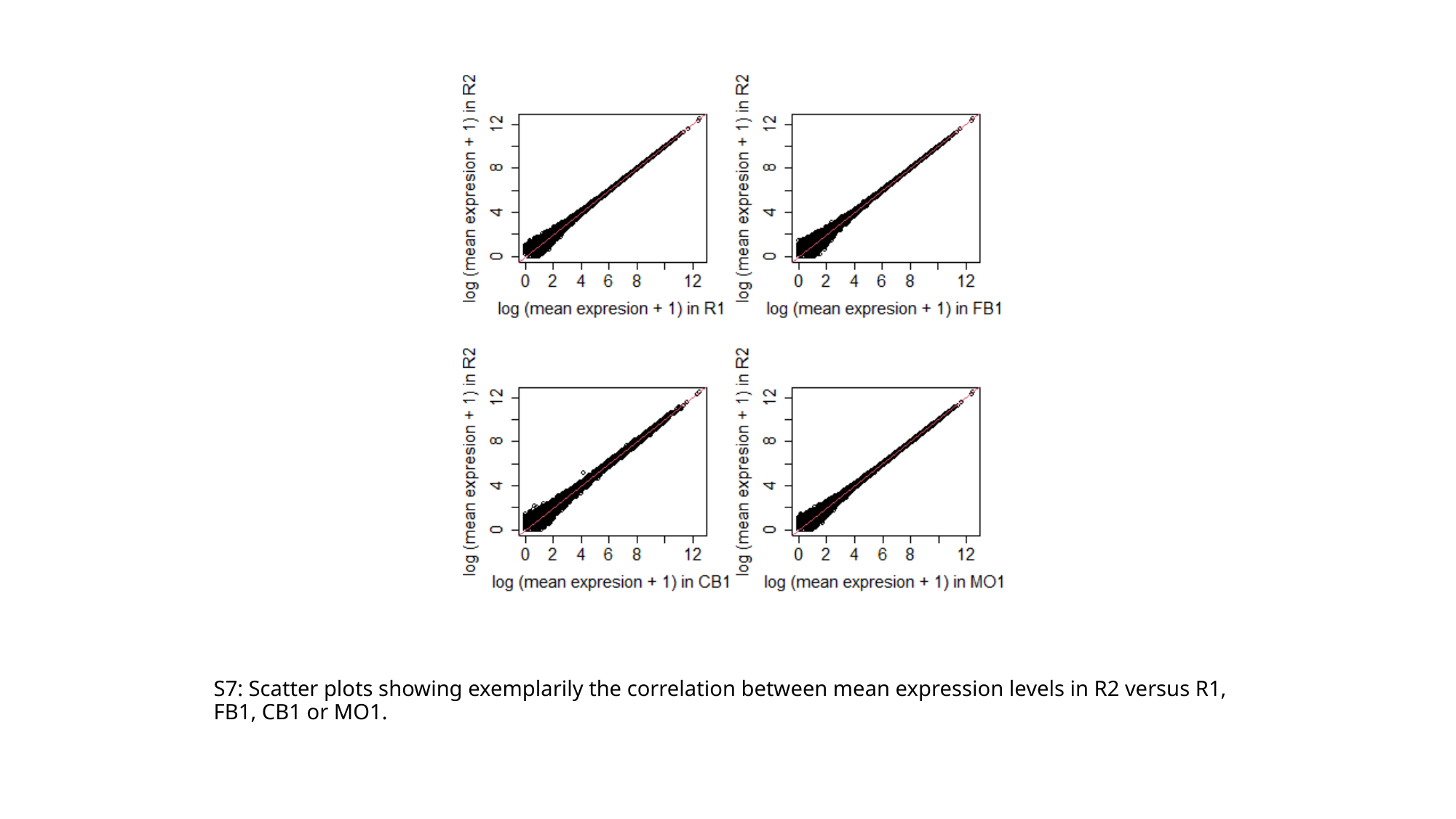

# S7: Scatter plots showing exemplarily the correlation between mean expression levels in R2 versus R1, FB1, CB1 or MO1.
