# Supplementary material for: A comparison of strategies for generating artificial replicates in RNA-seq experiments
Source: Sci Rep. 2022 May 3;12:7170. doi: 10.1038/s41598-022-11302-9 (PMC9065086; doi:10.1038/s41598-022-11302-9)
Supplement: Supplementary file 2 — Supplementary Information 2. [file 41598_2022_11302_MOESM2_ESM.zip › BATVDCdpi2RP1_S5_R2_001_fastqc.html]

BATVDCdpi2RP1\_S5\_R2\_001.fastq FastQC Report 

FastQC Report

Fr. 4 Feb. 2022  
BATVDCdpi2RP1\_S5\_R2\_001.fastq

## Summary

- Basic Statistics
- Per base sequence quality
- Per tile sequence quality
- Per sequence quality scores
- Per base sequence content
- Per sequence GC content
- Per base N content
- Sequence Length Distribution
- Sequence Duplication Levels
- Overrepresented sequences
- Adapter Content

## Basic Statistics

| Measure | Value |
| --- | --- |
| Filename | BATVDCdpi2RP1\_S5\_R2\_001.fastq |
| File type | Conventional base calls |
| Encoding | Sanger / Illumina 1.9 |
| Total Sequences | 38793345 |
| Sequences flagged as poor quality | 0 |
| Sequence length | 35-75 |
| %GC | 47 |

## Per base sequence quality

## Per tile sequence quality

## Per sequence quality scores

## Per base sequence content

## Per sequence GC content

## Per base N content

## Sequence Length Distribution

## Sequence Duplication Levels

## Overrepresented sequences

No overrepresented sequences

## Adapter Content

Produced by FastQC (version 0.11.8)
